# The efficacy of binary ethylenimine-inactivated vaccines of Gianyar-1/AK/2014 virulent strain in protecting chickens against Tabanan-1/ARP/2017 virulent Newcastle disease virus isolates

**DOI:** 10.14202/vetworld.2019.758-764

**Published:** 2019-06-08

**Authors:** Anak Agung Ayu Mirah Adi, I Nyoman Mantik Astawa, I Gusti Agung Arta Putra

**Affiliations:** 1 Laboratory of Veterinary Pathology, Faculty of Veterinary Medicine, Udayana University, Kampus Sudirman, Jalan PB Sudirman, Denpasar, Bali, Indonesia; 2 Laboratory of Veterinary Virology, Faculty of Veterinary Medicine Udayana University, Kampus Sudirman, Jalan PB Sudirman, Denpasar, Bali, Indonesia; 3 Laboratory of Animal Anatomy and Physiology, Faculty of Animal Husbandry, Udayana University, Kampus Bukit, Jimbaran, Badung, Bali, Indonesia

**Keywords:** genotype VII, inactivated vaccine, live vaccine, Newcastle disease virus

## Abstract

**Aim:**

This study aimed to prepare binary ethylenimine (BEI)-inactivated virulent Newcastle disease virus (NDV) vaccine and to examine their ability to induce a protective antibody response in commercial chickens.

**Materials and Methods:**

A virulent NDV field isolate Gianyar-1/AK/2014 was propagated in chicken-embryonated eggs and was then inactivated with BEI at a concentration of 4 mM. Three groups of chickens with low-level (2 log_2_ hemagglutination inhibition [HI] units) maternally derived antibodies against NDV were then immunized with the BEI-inactivated vaccine. A commercial live vaccine (LaSota strain) was used as positive control, and phosphate-buffered saline (PBS) was used as negative control. A challenge experiment with a virulent NDV of Tabanan-1/ARP/2017 was performed at 3 weeks post-vaccination.

**Results:**

At 2 weeks post-immunization, the mean titers of antibodies against NDV in serum samples of chickens immunized with 0.2 mL of BEI-inactivated NDV (Group I), with live commercial NDV vaccine (Group II) and with PBS (Group III) were 3±0.94 log_2_ HI units, 4.9±0.99 log_2_ HI unit, and 0.0±0.0 HI units, respectively. At week 3 post-immunization, the mean titers of the antibodies for the three groups were 5±1.09 log_2_ HI units, 6.9±0.32 log_2_ HI units, and 0.00 HI units, respectively. The antibody titer induced by inactivated NDV Gianyar-1/AK/2014 isolates examined at 2 and 3 weeks post-vaccination was still at a significantly (p<0.01) lower level as compared to those induced by commercial life vaccine. However, the challenge test with virulent NDV of Tabanan 1/ARP/2017 isolates showed that all immunized chickens (Group I and II) survived without exhibiting any clinical sign post-challenge with the protection rates of 100%, whereas all chickens injected with PBS (Group III) died with clinical signs of ND.

**Conclusion:**

This finding shows that the BEI-inactivated vaccines prepared using virulent NDV of Gianyar-1/AK/2014 strain was able to induce protective antibody response in chickens but still at a lower level than those induce by commercial live NDV vaccine.

## Introduction

Newcastle disease (ND) is a devastating poultry disease caused by a virulent strain of *Avian paramyxovirus* type 1 (APMV-1). The APMV-1, which is also known as ND virus (NDV), has a very wide genetic diversity but can genetically be classified into two distinct classes: Class I and II. Viruses of Class I are mostly found in wild waterfowls and generally consist of avirulent strains. The viruses of Class II are found in poultry and consist of both avirulent and virulent strains. Currently, there are 18 genotypes (genotype I-XVIII) in Class II [1]. The genotype VII is the most common genotype found as a causative agent of ND in Indonesia [2-6]. Generally, the genotype of NDV isolates found in the field differs to those of NDV strain the commonly used in the preparation of vaccine such as LaSota vaccine that belongs to genotype II [7]. Such genotype differences, which in turn lead to antigenic differences can affect the efficacy of the immune responses of host-induced by NDV vaccine in preventing infection. The antigenic difference can also be one contributing factor to the ND outbreak that still occurs frequently in vaccinated birds. Thus, the use of local virulent virus isolates as a vaccine candidate might be advantageous in terms of its high antigenic identity level to the virulent NDV causing ND outbreak in the field as both vaccine and field viruses belong to the same genotype.

Vaccination to prevent ND is routinely carried out in the endemic areas [8]. The types of vaccines and vaccinations schedules implemented vary depending on the potential threats, the virulence of the field viruses, and the immune status of the hosts. Vaccination to control ND in poultries is generally aimed to reduce or to eliminate the clinical disease, to reduce the amount of virus shedding, and to prevent death after infection with virulent NDV [9]. Vaccination with avirulent or low virulent live and inactive vaccines is implemented routinely to protect chickens from ND [10]. Although vaccination with inactive NDV vaccine is less common than those with live NDV vaccine, the inactive vaccine can be advantageous as it can be prepared using a wide range of NDV strains or genotypes, both using low and high virulent viruses [11]. At present, the inactive NDV vaccines available on the market are those contain viral antigens inactivated with formaldehyde (formalin) or β-propiolactone (BPL). The BPL inactivates the virus by alkylating nucleic acids (adenosine) and proteins (methionine, cysteine, and histidine) [12], while formaldehyde reacts with both nucleic acids and proteins [13] which cross-link both among proteins and between proteins and nucleic acids. As both BPL and formaldehyde react with proteins, their use as inactivating agents for preparation of vaccines can, to some extent, interfere with the antigenicity of the viral antigen.

Meanwhile, binary ethylenimine (BEI) is an aziridine compound that has been used to inactivate many viruses so that it can be utilized further for vaccine preparations [14,15]. BEI reacts with viral nucleic acids while maintaining the conformation and accessibility of the epitope to a much greater degree than formaldehyde and BPL [15]. Various viruses have been successfully inactivated using BEI for vaccine production, such as infectious bursal disease viruses [16], foot and mouth disease viruses [17], rabies virus [18-20], and Japanese encephalitis viruses [21].

To minimize the problem associated with the loss of antigenicity due to virus inactivation, an inactive virulent NDV as a candidate of new seed vaccine has been developed using virulent NDV Gianyar-1/AK/2014 isolate (genotype VII) inactivated with BEI. The immune responses and the protection rates of the BEI-inactivated vaccine were compared with live commercial vaccine in commercial chickens.

## Materials and Methods

### Ethical approval

This research has been officially agreed by the Ethical Commission for the Use of Animals in Research and Education of the Faculty of Veterinary Medicine, Udayana University, Indonesia, with approval number: 255a/KE-PH-Lit/VII/2016.

### Virus, viral propagation, purification, and inactivation

Two virulent NDV field isolates were used in this study. They were designated, respectively, as Gianyar-1/AK/2014 NDV isolate [5] which was used as a vaccine candidate and Tabanan-1/ARP/2017 isolate (GenBank accession number. MH 215997.1) which was used as a challenge virus. Each isolate was propagated in chicken-embryonated eggs (CEEs) and was retested for NDV before the experiment, as described below.

Both NDV isolates were propagated by inoculation into allantoic cavity of 9- to 10-day-old anti-NDV antibody-free CEEs obtained from Disease Investigation Center (DIC, Denpasar, Indonesia). The inoculated CEEs were monitored for 24-72 h to observe embryo death. Allantoic fluid of inoculated CEEs was harvested and centrifuged at 1000×g, for 30 min. The supernatant was collected and tested for the presence of NDV with hemagglutination (HA) and HI test. The test was performed according to the procedures as described by OIE [8]. To ensure that allantoic fluid contains only NDV HI test using anti-avian influenza (AIV) was performed using standard methods as described by Adi *et al*. [2]. Infective allantoic fluid-containing NDV was clarified by centrifugation at 1500×g for 10 min at 4°C, filtered with 0.4 µm filter, and kept in micro-tubes at −80°C for further use.

The presence of NDV in allantoic fluid was also confirmed by a reverse transcriptase polymerase chain reaction (RT-PCR) test using a pair of primers: F10s: 5′-GCAGCTGCAGGGATT GTGGT-3T and F10r: 510CTTTGAGC AGGA GGA TGTTG-3T. The primers amplified the F gene fragment of the NDV genome at the position of 4680-4990. The cycling time of the one-step RT-PCR reaction was performed according to those described by Putra *et al*. [5].

### Viral inactivation

The egg-derived NDV Gianyar-1/AK/2014 isolate was inactivated with 2-bromoethylamine hydrobromide as exemplified by Mondal *et al*. [18]. Briefly, the 2-bromoethylamine hydrobromide was dissolved in 0.5 NaOH with a final concentration of 0.6 M and incubated for 1 h at 37°C to convert the compound to BEI, an active compound that inactivates the virus by damaging the nucleic acid of the virus. BEI was then added to the NDV stock at the final concentration of 0.03 M, mixed thoroughly, and incubated overnight at 37°C. The antiviral effect of BEI was neutralized by adding sodium thiosulfate (final concentration of 2%). Complete inactivation of the virus was confirmed by inoculation the BEI-treated virus in 10-day-old anti-NDV antibody-free CEEs for three passages. Inactivated virus was then stored at −20°C, until use. To produce the vaccine, the inactivated viral antigens were mixed with alum adjuvant (Alhydrogel 2%, InvivoGen, USA) at a ratio of 1:1 (V/V) before being injected in chickens.

### Western blotting

Western blotting analysis was performed to confirm the purity of the NDV isolates using anti-F2 protein of NDV [22] following the procedure as previously described [20]. Briefly, two samples of active isolate NDV Gianyar-1/AK/2014 and Tabanan-1/ARP/2017 and BEI-inactive Gianyar-1/AK/2014 were subjected to sodium dodecyl sulfate-polyacrylamide gel electrophoresis. The separated proteins were transferred to nitrocellulose membrane for 2 h in a wet tank mini transblot system (Bio-Rad, Hercules, CA, U.S.A.) using a previously described carbonate-bicarbonate transfer buffer system. The presence of F protein was visualized by adding mAb against F2 protein of NDV, biotinylated anti-mouse IgG (KPL, USA), streptavidin-alkaline phosphatase (KPL, USA), and BCIP/NBT (KPL, USA).

### Experimental chickens

A total of 100 day-old chicks (DOCs) of ISA brown strain obtained from a commercial hatchery (P.T. Charoen Pokphand Jaya Farm, Jembrana, Bali, Indonesia) were used in this study. All DOCs were brooded together until 2 weeks in a cage with heating facilities. At 2 weeks old, each chick was tagged with cable ties in their leg and with a number to identify the individual chickens. Serum samples were then collected from each chick at 2 and 3 weeks old for the measurement of maternally derived antibody (MDA) against NDV using HI test. Chickens with low MDA titers (2 log_2_ HI units) were selected for the experiments, and those with MDA titers higher than 2 log_2_ HI units were excluded.

### Experimental design

#### Vaccination and challenge test

Thirty chickens with MDA titer of 2 log_2_ HI unit were divided equally into three groups, Group I, II, and III, and each group was introduced into three different pens in a separated room. Group I was immunized with 0.2 mL BEI-inactivated vaccine Gianyar-1/AK/2014, containing approximately 2^8^ HA units. Group II was injected with LaSota commercial vaccine Medivac ND LaSota (Medion, Bandung, Indonesia) at the doses of 10^7^ EID_50_ and Group III was injected with phosphate-buffered saline (PBS), all by intramuscular injection. The antibody titers post-injection were monitored twice, at 2 and 3 weeks post-injection. Challenge test to all chicken was performed at 3-week post-vaccination, using virulent NDV Tabanan-1/ARP/2017 isolate at dose 0.2 mL of 10^5^ TCID_50_ orally per chicken. Observations for clinical signs and death were carried out for 28 days post-challenge. Serum samples from survived chicken were collected at 1-week, 2-week, 3-week, and 4-week post-challenge for the HI test. Virus isolation to determine viral shedding was carried out on the 2^nd^- and 5^th^-day post-challenge from the cloacal swab pool. Virus isolation was also carried out from tissue samples of dead chickens post-challenged.

### Examination of morbidity and mortality rate, histopathology, and immunohistochemistry (IHC)

Morbidity and mortality rates were examined by counting and comparing the numbers of chickens died after challenge test in the three treatment groups. Post-challenge dead chickens were then necropsied, and tissue samples of the brain, trachea, thymus, lung, heart, intestine, spleen, kidney, and bursa of Fabricius were collected for histopathology and for IHC staining using anti-NDV F2 monoclonal antibody as described previously by Adi *et al*. [2] and Astawa and Adi [22].

### Statistical analysis

The data of mean titers of antibodies against NDV in the three groups of chickens were analyzed with t-test. However, morbidity, mortality, clinical signs, and pathological features of all groups were compared descriptively.

## Results

### The titers and the purity of NDV isolates

**Figure-1:**
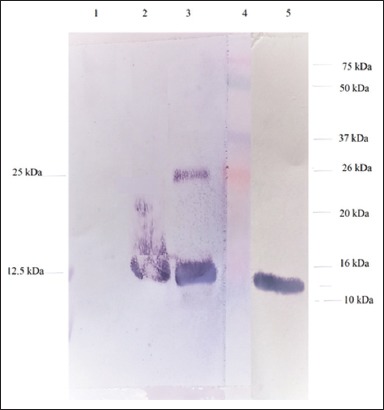
Profiles of F2 proteins of Gianyar-1/AK/2014 (native and binary ethylenimine [BEI]-inactivated) and Tabanan-1/ARP/2017 isolates analyzed by Western blotting using anti-Newcastle disease virus F2 mAb. (Line 1, 2, 3, and 5): Normal allantoic fluid, BEI-inactivated Gianyar-1/AK/2014), active Gianyar-1/AK/2014, and Tabanan-1/ARP/2017, respectively. Line 4: prestained protein standard marker.

The two NDV isolates used in this study were propagated in anti-NDV antibody-free chicken CEEs. The allantoic fluid harvested from the inoculated CEEs was confirmed serologically to contain only NDV. HI test using anti-AIV antibody showed that AIV was not detected in the harvested allantoic fluid. Test results with RT-PCR with specific F gene primers also ascertained that the allantoic fluid contains NDV. The presence of NDV viral proteins in Gianyar-1/AK/2014 (native and BEI-inactivated) and in Tabanan-1/ARP/2017 isolates was confirmed by Western blotting using anti-NDV F2 mAb. The F2 protein was identified as 12.5 kDa protein band, and additional of 25 kDa protein was also detected in active Gianyar-1/AK/2014 isolate (Figure-1).

### Inactivation effectiveness of BEI against NDV

To ascertain the complete inactivation of the virus, the BEI-treated Gianyar-1/AK/2011 was inoculated into allantoic cavity of 10-day-old anti-NDV antibody-free CEEs. All CEEs injected with BEI-treated viruses still alive after 7 days following three passages, and no residual active NDV was detected by HA test.

### Profiles of anti-NDV maternal antibodies

**Figure-2:**
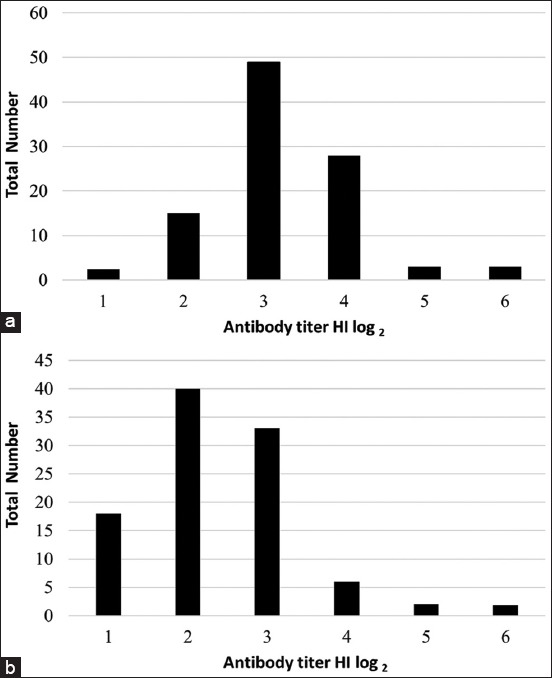
Profile of Newcastle disease virus (NDV) maternally derived antibody of commercial layer chicken at 2 weeks old (a) and 3 weeks old (b). Note: Antibody titers >3 log_2_ HI units, which are believed to be able to protect against NDV.

In this experiment, the MDA against NDV was determined at the times when the chickens were at 2 and 3 weeks old. The dynamics of MDA level were as follows. At the age of 2 weeks, a total of 17 (17%) out of 100 chickens already had MDA level ≤2 log_2_ HI units (Figure-2a), and at the age of 3 weeks, a total of 58 (58%) out of 100 chickens already had low MDA level ≤2 log_2_ HI units (Figure-2b).

### Response of chickens to vaccination

**Figure-3:**
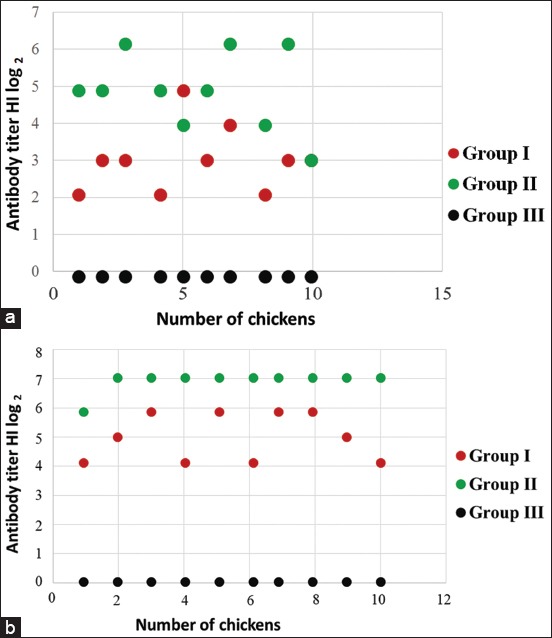
Antibody titers profile at 2 weeks (a) and 3 weeks (b) after vaccination. Group I was vaccinated with inactivated Gianyar-1/AK/2014, Group II was vaccinated with LaSota life vaccine, and Group III was control as injected with phosphate-buffered saline.

Data related to antibody titers against NDV in the sera samples of chickens post-vaccinations are presented in Figure-3. At 2 weeks post-vaccination, the mean titers of antibodies in chickens receiving 0.2 mL BEI-inactivated NDV (Group I), commercial live NDV vaccine (Group II), and non-vaccinated chickens (Group III) were 3±0.94 log_2_ HI unit, 4.9±0.99 log_2_ HI unit, and 0.0±0.0 HI unit, respectively (Figure-3a). The mean anti-NDV antibody titers at 3 weeks post-vaccination for three groups were 5±1.09 log_2_ HI units, 6.9±0.32 log_2_ HI units, and 0.00 HI units, respectively (Figure-3b).

### Response of chickens to challenge

After the challenge, all chickens of Group I and II survived and remained free from clinical signs of infection, whereas all of those in Group III died with clinical signs of ND.

The clinical signs of ND, such as depression, trembling, ruffled feathers, and greenish diarrhea were observed starting from day 2 post-challenge, with varying severity. Five chickens found died at the 4^th^-day post-challenge and the rest on the next day. Virus isolation from the cloacal swab of vaccinated chickens at day 2 and 5 post-challenge (p.c) was negative. However, the virus was isolated from unvaccinated chicken (Table-1).

**Table-1:** Clinical sign, viral shedding, viral re-isolation from dead chicken, and morbidity and mortality rate.

Group	Viral shedding		Morbidity rate	Mortality rate	Clinical signs observed
			
	2^nd^ pc	5^th^ pc	(a/b)	(c/d)	
I	Negative	Negative	0/10	0/10	No clinical sign
II	Negative	Negative	0/10	0/10	No clinical sign
III	Positive	Positive	10/10	10/10	3 days post-challenge: Depression, trembling, ruffled feathers, and greenish diarrhea. Five chicken died at day 4 and the rest at day 5 pc

After the challenge test, all chickens of Group I and II remained free from clinical signs of infection; however, all naive chicken (Group III) had positive clinical signs, and finally, all chickens died. pc=Post-challenge

**Figure-4:**
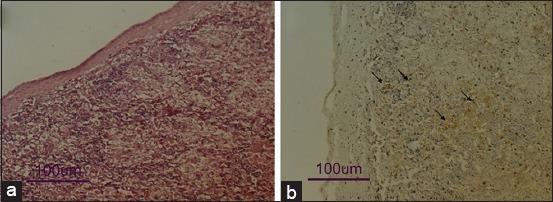
(a) microscopic changes of the spleen with hemorrhage and mark necrosis, especially around white pulp (Hematoxylin-Eosin staining). (b) The Newcastle disease virus-antigen-positive cell surrounding necrotic area; Immunohistochemical staining, with Diaminobenzidine substrate and Mayer’s Hematoxylin counterstain.

The prominent macroscopic changes found in chickens died post-challenge were hemorrhage and edema. The microscopic changes found were hemorrhage, edema, necrosis, and infiltration of inflammatory cells in various organs. IHC staining using anti-NDV monoclonal antibodies found the NDV antigen in the cells of various tissue samples, except the brain. The cells containing NDV antigen with the highest intensity were found in the spleen (Figure-4).

### Dynamic of antibody titer post-challenge

**Figure-5:**
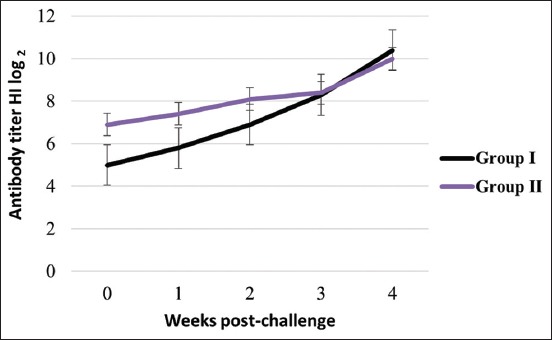
Dynamic of Newcastle disease virus hemagglutination inhibition titer antibody post-challenge. Group I was vaccinated with inactivated vaccine Gianyar-1/AK/2014 and Group II with LaSota life vaccine.

In another continuing work, two groups of chickens that survived post-challenge were monitored for their NDV antibody dynamics, starting from 1 to 4 weeks post-challenge. HI test showed that the titers of anti-NDV increase significantly after challenge (Figure-5).

## Discussion

In this study, ISA brown commercial chicken was used as an experimental animal, instead of specific pathogen-free chicken to mimic the real-field conditions, as this strain of chickens is a common layer raised by farmers in Indonesia. MDA against NDV is one important factor that can affect the immune response of chickens to vaccination against NDV. Theoretically, the MDA level against NDV decline gradually, and by 3 weeks old, it will be mostly below the minimum protective level. In this study, however, HI test showed that by 3 weeks old, 42% of the chickens had MDA above the minimum protection level ≥3 log_2_ HI units (Figure-2b). The chickens with MDA above the minimum protective level were excluded from this study such that MDA level can interfere with immune response of chickens to NDV vaccination. Although MDA can serve as passive protection against infection in the early age of life, high level of MDA can suppress B-cell response, which can result in low immune responses to avian pathogen [23,24]. The MDA can affect immune response following vaccination against diseases, such as ND and AI [25]. As reported previously, MDA can interfere with vaccination for up to 3 weeks after hatching [26], and therefore, only chickens with antibody level 2 log_2_ HI units were used as experimental animals in this study.

In this study, BEI was selected as an agent to inactivate the virus, and BEI inactivates viruses by binding mainly to guanine nucleotide or to small amount of adenine nucleotide in RNA or DNA molecules to form alkylated nucleotides which inactivate virus without interfering with the antigenicity of the viral antigen [15]. Full inactivation of the virulent NDV isolate was further confirmed by inoculation of the inactivated virus in CEEs. Following three passages in CEEs, the embryo in CEEs survived for 7 days, and no virus was detected in allantoic fluid of the inoculated CEEs by HA test. This shows that BEI is a potent virus-inactivating agent which can inactivate virulent NDV after incubation at 37°C for overnight as suggested by the previous study of Mondal *et al*. [18].

Immunization of chickens with BEI-inactivated virus also showed the inactivated virus was immunogenic. The presence of NDV antigen in G1/AK/2014 and Tabanan-1/ARP/2017 strains was confirmed by Western blotting analysis using anti-F2 antibodies (Figure-1). The mean titers of antibody against NDV in the serum samples of chicken immunized with BEI-inactivated Gianyar 1/AK/2014 NDV isolate at 3 weeks post-vaccination were 5 log_2_ HI units, which was lower as compared to those of chicken vaccinated with commercial live vaccine (6.9 log_2_ HI units). Although the antibody titers against NDV in chickens immunized with BEI-inactivated virulent NDV isolate was slightly lower than those immunized with live commercial vaccine, antibody response induced by both vaccines fully protected the chickens from challenge virus (Table-1). This result is in line with previous study that antibody titer 5 log_2_ HI units could protect chicken from NDV infection [27].

Both the Gianyar-1/AK/2014 and Tabanan-1/ARP/2017 isolates are virulent NDV isolates from genotype VII [5]. The antigenic identity levels between these two field isolates are potentially higher as compared to those between the two field isolates and the commercial NDV vaccine strain used for vaccination which can be advantageous of using local virulent NDV isolate as a candidate of vaccines. Thus, the use of local virulent NDV isolate as a candidate of vaccine is expected to be able to reduce the occurrence of DV outbreaks in the vaccinated birds.

The results of this study also show that both BEI-inactivated vaccine (Group I) and live vaccine (Group II) have equal protection rate of 100% as all immunized chickens survived after challenge with Tabanan 1/ARP/2017 (Table-1). It seems that the problem associated with vaccination failure is not entirely due to the lack of protection of immune response induced by vaccine but can be due to miss management of vaccinations, such as poor handling or administration of vaccines and improper vaccination schedules.

One of many factors that can contribute to the vaccination failure was the presence of MDA. In this study, chickens at 3 weeks of age were still found to have high titers MDA (Figure-2). The high titers of MDA can interfere with vaccination results as it can inhibit the immune response due to vaccination [28]. Chickens with MDA of above protective level were then excluded from this study and can be included in the next study to investigate the possible role of MDA in interfering with vaccination both with inactivated and live vaccines.

When vaccination of chickens with NDV vaccines conducted properly, and chickens were given enough time to have an adequate immune response, they will be able to prevent the infection and will show no signs of disease. As reported by Cornax *et al*. [29] and Miller and Koch [30], the application of vaccines is very important in controlling ND disease in poultry. However, field condition may not be same as experimental conditions where under the field condition, chickens were vaccinated at the times when the titers of MDA had not been declining to an unprotective level (HI ≤2 log_2_ HI units). In addition, under the field conditions, the infection with virulent NDV can occur at any times after vaccination, instead of 3 weeks after vaccination under experimental conditions.

In post-challenge infection, all vaccinated chickens survived without exhibiting any clinical signs or dead, whereas all unvaccinated chickens died with the clinical signs and pathological changes of ND (Table-1). In addition, NDV antigen was only detected in chickens died from ND (unvaccinated chickens) (Figure-4) and was not detected of chickens survived from challenge test (vaccinated chickens). HI test showed that the antibody titer post-challenge increased gradually in vaccinated chickens survived from challenge test (Figure-5). The vaccination with both BEI-inactivated and live commercial vaccines appeared to have primed chickens to NDV antigen, which enables them to neutralize the virus, and therefore survived from the infecting virus, as well as induced higher antibody titers after infection with virulent NDV.

## Conclusion

Virulent inactive vaccine of genotype VII Gianyar-1/AK/2014 has been developed in laboratory condition, and its efficacy was compared with commercial ND vaccine. It was found that although this inactive vaccine induced a lower antibody response than live commercial vaccine, it could fully protect the chickens from NDV virulent type genotype VII at equal level as induced by live vaccine.

## Authors’ Contributions

AAAMA executed the work (designing the study, RT-PCR work, pathological examination, viral isolation, and drafting of manuscript); INMA participated in serological work, viral inactivation, western blotting, vaccine preparation, tissue culture work, and checking the manuscript; IGAAP participated in handling experimental chicken, blood collection, and sera preparation. All authors read and approved the final manuscript.
